# Haptoglobin genotype is a determinant of survival and cardiac remodeling after myocardial infarction in diabetic mice

**DOI:** 10.1186/1475-2840-8-29

**Published:** 2009-06-02

**Authors:** Roy Asaf, Shany Blum, Ariel Roguin, Shiri Kalet-Litman, Jad Kheir, Avi Frisch, Rachel Miller-Lotan, Andrew P Levy

**Affiliations:** 1 Department of Anatomy and Cell Biology, Rappaport Faculty of Medicine, Technion Israel Institute of Technology, Haifa, Israel; 2 Department of Cardiology, Rambam Health Care Campus, Haifa, Israel

## Abstract

**Background:**

We have recently demonstrated in man that a functional allelic polymorphism in the Haptoglobin (Hp) gene plays a major role in determining survival and congestive heart failure after myocardial infarction (MI). We sought to recapitulate the effect of Hp type on outcomes and cardiac remodeling after MI in transgenic mice.

**Methods:**

The Hp 2 allele exists only in man. Wild type C57Bl/6 mice carry the Hp 1 allele with high homology to the human Hp 1 allele. We genetically engineered a murine Hp 2 allele and targeted its insertion by homologous recombination to the murine Hp locus to create Hp 2 mice. Diabetes Mellitus (DM) was induced with streptozotocin. MI was produced by occlusion of the left anterior descending artery in DM C57Bl/6 mice carrying the Hp 1 or Hp 2 allele. MI size was determined with TTC staining. Left ventricular (LV) function and dimensions were assessed by 2-dimensional echocardiography.

**Results:**

In the absence of DM, Hp 1-1 and Hp 2-2 mice had similar LV dimensions and LV function. MI size was similar in DM Hp 1-1 and 2-2 mice 24 hours after MI (50.2 ± 2.1%and 46.9 ± 5.5%, respectively, p = 0.6). However, DM Hp 1-1 mice had a significantly lower mortality rate than DM Hp 2-2 mice 30 days after MI (HR 0.41, 95% CI (0.19–0.95), p = 0.037 by log rank). LV chamber dimensions were significantly increased in DM Hp 2-2 mice compared to DM Hp 1-1 mice 30 days after MI (0.196 ± 0.01 cm^2 ^vs. 0.163 ± 0.01 cm^2^, respectively; p = 0.029).

**Conclusion:**

In DM mice the Hp 2-2 genotype is associated with increased mortality and more severe cardiac remodeling 30 days after MI.

## Background

The increasing burden of cardiovascular diseases (CVD) has been unquestionably linked with the growing epidemic of diabetes mellitus (DM). CVD is the leading cause of death in individuals with DM [1]. Conventional risk factors, such as smoking, hypertension, and hyperlipidemia provide a substantial, yet, limited explanation for the increased risk of CVD in DM [2]. Recent studies have further underscored that hyperglycemia is a necessary but not sufficient condition for the development and progression of diabetic CVD [3]. Several polymorphic genetic loci have recently been identified that may serve as important determinants of the susceptibility to and rate of progression of cardiovascular complications in DM [4-9]. One such polymorphic locus is that encoding the gene for Haptoglobin (Hp) [10,11].

Hp is an acute phase serum protein whose main known function is binding and scavenging of free hemoglobin through the liver or circulating monocytes [12-14]. In man there exist a functional polymorphism in the Hp gene with two common alleles denoted 1 and 2 that result in three possible genotypes. The Hp protein produced by the Hp 1 and Hp 2 alleles differs markedly both in its structure and function. In a post hoc analysis of clinical trials comprising several thousand patients we have found that the Hp 2-2 genotype is an independent risk factor for cardiovascular complications in DM [11,15-17].

In particular we have demonstrated that the Hp genotype is a determinant of congestive heart failure (CHF) and death after MI in man. Individuals with the Hp 2-2 genotype were found to have a 7 fold greater mortality and 4 fold greater incidence of CHF 30 days after MI as compared to Hp 1-1 DM individuals after MI [16].

In this study we sought to determine if this association between the Hp genotype and DM on outcomes and cardiac remodeling after MI could be recapitulated in transgenic mice.

## Methods

### Animals

All protocols used in these studies were approved by the Technion Faculty of Medicine Animal Care and Use Committee. The Hp 2 allele exists only in man. Wild type C57Bl/6 mice carry only the Hp 1 allele with a high homology to the human Hp 1 allele [18].

We genetically engineered a murine Hp 2 allele and targeted its insertion by homologous recombination to the murine Hp locus to create Hp 2-2 mice as previously described [19].

### Diabetes

DM was induced at 2–3 month of age by IP injection of 50 mg/kg of streptozotocin (STZ) on 5 consecutive days. Blood glucose levels were monitored once a week with DM being defined as a consistent blood glucose level higher than 200 mg/dl.

### MI

Myocardial infarction was produced using a modification of a previously described ischemia-reperfusion model [20]. Briefly, mice were anesthetized with a mixture of ketamine (150 mg/kg) and xylazine (9 mg/kg) and the trachea intubated and connected to a small rodent respirator (MiniVent 845, Harvard Apparatus). The chest was opened by a left lateral thoracotomy and after removal of the pericardium a ligation of the left anterior descending coronary artery (LAD) was done using 7/0 prolene non-absorbable suture thereby permanently occluding blood flow to the myocardium distal to the ligature. The mouse was then weaned off from ventilation and allowed to awaken. The procedure was done under vision with a stereoscopic zoom microscope (Nikon SMZ800). Mice were studied for up to 30 days after the MI and then sacrificed.

### Infarct size

Hearts were harvested 24 h after MI. After the right ventricle was removed the left ventricle was cooled for 5–10 minutes and then cut into five 3 mm thick slices along its short axis. All slices were weighed and then incubated at 37° in 1% 2,3,5 – triphenyltetrazoliumchloride (TTC) in phosphate saline buffer (pH 7.0) for 20 minutes. After incubation in TTC the slices were photographed and the images were used for planimetric analysis to determine relative infarct size of the total LV area.

### Echocardiography

Trans-thoracic echocardiography was performed with a commercially available echocardiography system equipped with a 15-MHz phased-array linear transducer (General Electric) by a single experienced operator who was blinded to the Hp genotype and to the time point at which the data was recorded. Animals were sedated with a low dose mixture of ketamine (50 mg/kg) and xylazine (3 mg/kg). Animals underwent three echocardiography examinations. A baseline examination was preformed 24–48 hours prior to the operation, a second examination 4 days after the operation and a third examination 30 days after the operation.

We measured maximal left ventricular end-diastolic diameter (LVEDd) and area (LVEDa) and minimal LV end-systolic diameter (LVESd) and area (LVESa) in 2-D mode imaging. Fractional shortening (FS), as a measure of systolic function was calculated as: FS (%) = [(LVEDd-LVESd)/LVEDd] × 100. For the analysis we averaged measurements of 3 consecutive cardiac cycles.

### Statistical analysis

Groups were compared using the Student *t *test. A p value of < 0.05 was considered statistically significant.

## Results

### Baseline characteristics of mice used in this study

After 6 weeks of DM, Hp 1-1 or Hp 2-2 mice were subjected to either a MI or a sham procedure. Table 1 presents baseline characteristics of the four groups (Hp 1-1 MI, Hp 1-1 sham, Hp 2-2 MI, Hp 2-2 sham) taken on the same day and prior to the surgical procedure (coronary ligation or sham procedure). Baseline characteristics were similar between MI and sham groups of the same genotype.

**Table 1:** Baseline characters of diabetic mice subjected to MI\Sham procedure

	Hp 1-1			Hp 2-2		
	MI	Sham	P value	MI	Sham	P value
**Age (days)**	132(14)	130(9)	0.73	137(12)	129(14)	0.051
**Diabetes duration (days)**	44(4.6)	44(4.4)	0.76	45(5.4)	42(4)	0.058
**Body weight (g)**	21.65(2.3)	21.8(2.7)	0.82	21.2(3.5)	21.7(3.2)	0.61
**Females (%)**	34.8	16.7	0.19	33.3	22.2	0.41
**Blood glucose (mg/dL)**	456(117)	532(51)	0.02	511(111)	500(111)	0.74

Numbers are mean value (S.D.). N = 23 and 18 for Hp 1-1 (MI and sham, respectively) and n = 30 and 18 for Hp 2-2 (MI and sham, respectively).

### Myocardial infarction size is similar in Hp 1-1 and Hp 2-2 mice

**Figure 1:**
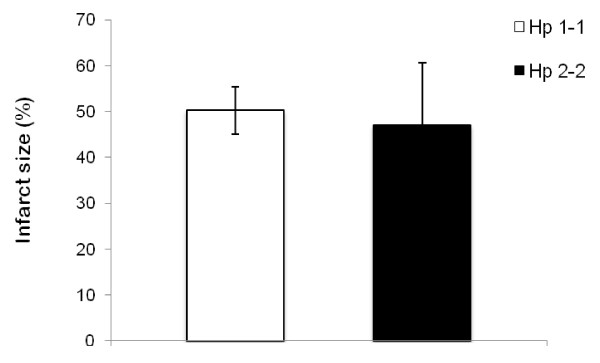
**MI size is similar in Hp 1-1 and Hp 2-2 mice 24 hours after MI**. Data shown is average ± S.D. n = 6 for Hp 1-1 and n = 7 for Hp 2-2. p = 0.6.

Figure 1 demonstrates infarct size in Hp 1-1 or Hp 2-2 DM mice 24 hours after complete LAD occlusion. MI size was calculated as infarct area relative to total LV area and adjusted to heart section weight. We found no difference in MI size between Hp 1-1 and Hp 2-2 DM mice (50.2 ± 2.1% and 47.0 ± 5.6%, respectively; p = 0.6).

### Survival is decreased after MI in Hp 2-2 DM mice

**Figure 2:**
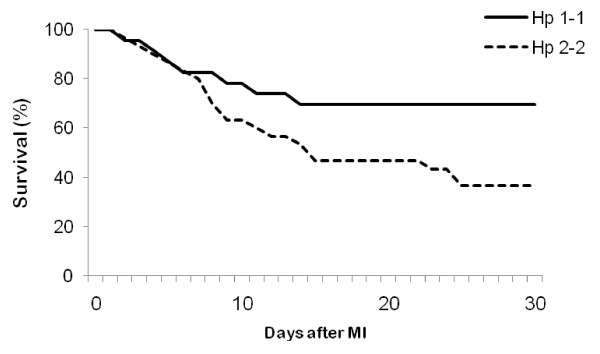
**30 day survival after MI is increased in Hp 1-1 mice as compared to Hp 2-2 mice**. N = 23 for Hp 1-1 and n = 30 for Hp 2-2. HR 0.41, 95% CI (0.19–0.95), p = 0.037 by log rank.

In spite of the similar MI size found in Hp 1-1 and Hp 2-2 DM mice, Hp 1-1 mice had a significantly higher survival rate during the 30 days after MI compared to Hp 2-2 mice (69.57% vs. 36.67%, respectively; HR 0.41, 95% CI (0.19–0.95), p = 0.037 by log rank) (Figure 2). There was no difference in survival between Hp 1-1 and Hp 2-2 sham operated mice (83.33% vs. 72.22%, respectively; p = 0.33).

### Left ventricular area is markedly increased in Hp 2-2 DM mice after MI

**Figure 3:**
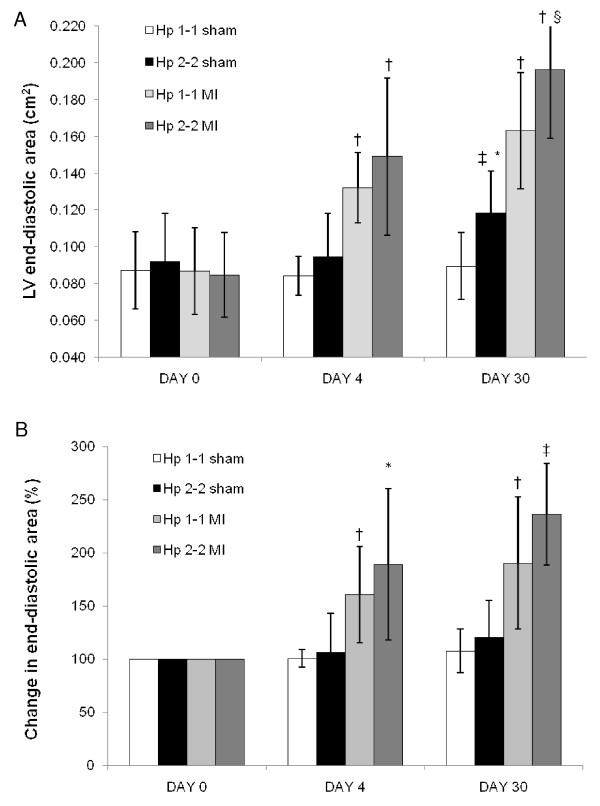
**Left ventricle end-diastolic area in DM mice after MI\Sham procedure**. Values are mean ± S.D. N = 22, 20, and 15 in Hp 1-1 mice (for day 0, 4, and 30 respectively). N = 29, 26, and 9 in Hp 2-2 mice (for day 0, 4, and 30 respectively). A – LVEDa. * p = 0.010 vs. 'DAY 0'; † p < 0.0001 vs. 'DAY 0'; §p = 0.012 vs. Hp 1-1; ‡ p = 0.0013 vs. Hp 1-1. B – Relative change in LVEDa compared to 'Day 0'. † p = 0.0001 vs. sham; ‡ p < 0.0001 vs. sham; * p = 0.002 vs. sham.

Mice in all four groups underwent three echocardiography examinations as detailed in Methods. At day 0, before any surgical intervention, LVEDa was similar between all four groups. In both Hp genotypes LVEDa was significantly increased at day 4 and day 30 after MI as compared to day 0 (p < 0.0001 at day 30 for Hp 1-1 and for Hp 2-2) (Figure 3A). However, LVEDa was significantly larger in Hp 2-2 mice as compared to Hp 1-1 mice 30 days after MI (0.196 cm^2 ^vs. 0.163 cm^2^, respectively, p = 0.029) (Figure 3A).

The net change in LVEDa 30 days after MI was 0.11 cm^2 ^and 0.07 cm^2 ^for Hp 2-2 and Hp 1-1, respectively (p = 0.027).

**Figure 4:**
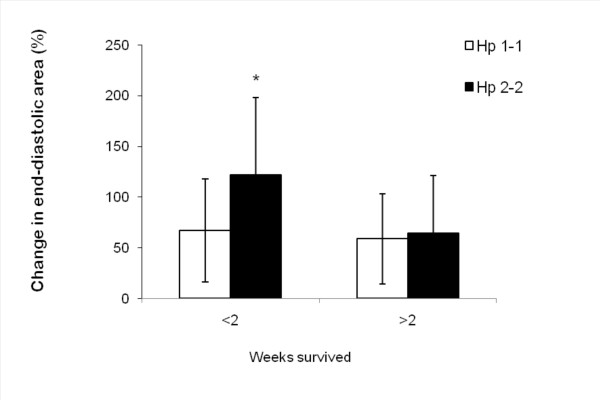
**Change in LV end-diastolic area 4 days after MI, segregated by Hp genotype and survival**. Values are mean ± S.D. N = 14 and 15 for Hp 1-1 mice (under and over 2 weeks, respectively). N = 11 and 5 for Hp 2-2 mice (under and over 2 weeks, respectively). * p = 0.044 vs. Hp 2-2 that survived more than 2 weeks.

In a sub-analysis we found that only in mice with the Hp 2-2 genotype was there a relationship between the increase in LVEDa four days after MI and early mortality (death within two weeks of the MI procedure). The relative change in LVEDa four days after MI was 121.35% in Hp 2-2 mice which died within two weeks of the MI (8.7 days on average) vs. 64.32% in Hp 2-2 mice which lived more than two weeks after the MI (27 days on average), p = 0.044. This phenomenon of an increased LVEDa in mice who died prematurely was not found in Hp 1-1 mice (Figure 4).

### Left ventricle function after MI\sham in DM

**Figure 5:**
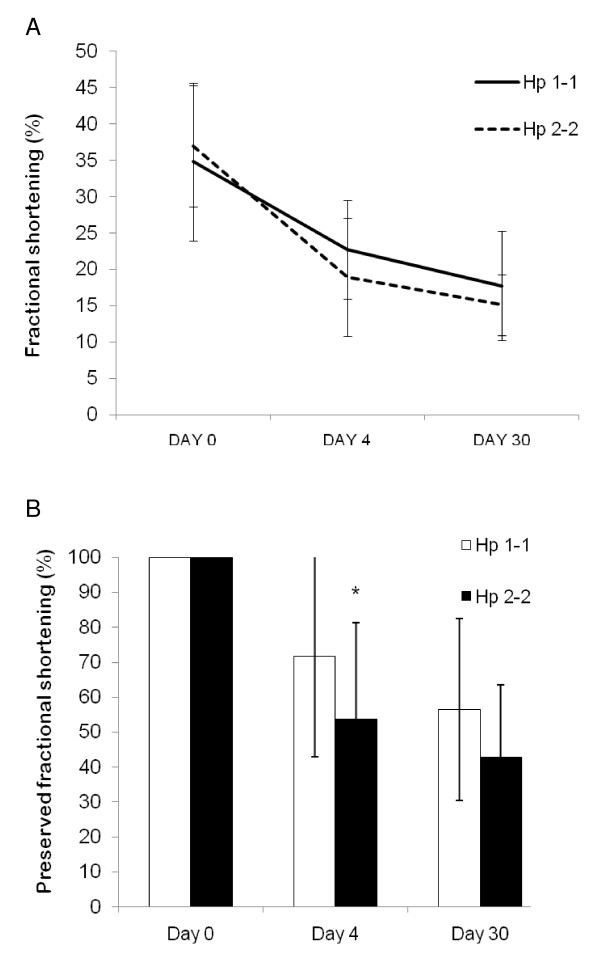
**Left ventricle function in DM mice after MI**. Values are mean ± S.D. N = 22, 20, and 15 in Hp 1-1 mice (for day 0, 4, and 30 respectively). N = 29, 26, and 9 in Hp 2-2 mice (for day 0, 4, and 30 respectively). A – Left ventricle function after MI. p = 0.42 at day 0, 0.1 at day 4, and 0.34 at day 30. B – Relative change in FS after MI compared to day 0. * p = 0.039 vs. Hp 1-1.

LV function was expressed by fractional shortening (FS) as described in Methods. The baseline FS was similar in Hp 1-1 and Hp 2-2 mice before MI. LV function was non-significantly better in Hp 1-1 mice as compared to Hp 2-2 mice at day 4 and day 30 after MI (Figure 5A); Moreover, in the Hp 2-2 group, 52% of the mice had at least a 50% reduction in FS 30 days after MI compared to 25% of the mice in the Hp 1-1 group (p = 0.066).

## Discussion

In this study we have demonstrated that Hp 2-2 DM mice have increased mortality and more severe cardiac remodeling after MI as compared to Hp 1-1 DM mice. These data therefore appear to recapitulate the association between Hp type and MI outcomes which we have previously found in man [16].

These results cannot be explained by differences in infarct size between the Hp 1-1 and Hp 2-2 mice. Prior studies showing an effect of Hp genotype on myocardial injury were done in an ischemia-reperfusion model using a reversible closure of the LAD while in the present study we have used a fixed occlusion of the LAD [20].

It is not clear why Hp 2-2 DM mice had a higher mortality rate compared to Hp 1-1 DM mice. Hp 2-2 DM mice which died within 14 days after MI had significantly more severe cardiac enlargement as compared to those Hp 2-2 mice which completed the study protocol and lived 30 days. Early mortality of more severely remodeled Hp 2-2 DM hearts may have been the reason why we were not able to demonstrate a significant decline in LV function in the Hp 2-2 DM mice.

Hp is expressed in the arterial wall and is involved in arterial remodeling by mediating ECM breakdown by inhibiting the activity of gelatinases and promoting cell migration [21]. This finding may apply to myocardial tissue as well. Hp has been shown to be increased in myocardial interstitial fluid after myocardial ischemia and regulates coronary collateralization after repetitive coronary occlusion [22]. It is possible then, that these functions of Hp may be genotype dependent and therefore affect early phase remodeling after MI.

## Conclusion

In this study we have recapitulated in a transgenic mice model the epidemiological association we have found in man between Hp genotype, death and cardiac remodeling. Therefore, this novel transgenic mouse model may serve as a platform to study the basic mechanisms that underlie cardiac remodeling which are Hp genotype dependent and to search for interventions aimed to modulate this process and attenuate the rapid progression of heart failure and decrease the high mortality rate after MI in Hp 2-2 DM individuals which make up 40% of the DM community.

## Competing interests

The authors declare that they have no competing interests.

## Authors' contributions

RA and AR carried out the ECHO measurements. RA and SB carried out the surgical procedure and participated in tissue processing. RML carried out the care of the animals before and after surgery. JK, AF, and SKL participated in tissue processing and in the ECHO measurements. APL and RA wrote the manuscript and designed the experiments. All authors read and approved the final manuscript.
